# Progression of arterial stiffness is associated with changes in bone mineral markers in advanced CKD

**DOI:** 10.1186/s12882-017-0705-4

**Published:** 2017-09-04

**Authors:** Rathika Krishnasamy, Sven-Jean Tan, Carmel M. Hawley, David W. Johnson, Tony Stanton, Kevin Lee, David W. Mudge, Scott Campbell, Grahame J. Elder, Nigel D. Toussaint, Nicole M. Isbel

**Affiliations:** 1 Department of Nephrology, Sunshine Coast University Hospital, PO Box 5340, Sunshine Coast, Birtinya, MC QLD 4560 Australia; 2 Faculty of Medicine, The University of Queensland, Brisbane, Australia; 3 Department of Nephrology, The Royal Melbourne Hospital (RMH), Melbourne, VIC Australia; 4 Department of Medicine (RMH), The University of Melbourne, Parkville, VIC Australia; 5 Translational Research Institute, Brisbane, Australia; 6 Department of Nephrology, Princess Alexandra Hospital, Brisbane, Australia; 7 Department of Cardiology, Sunshine Coast University Hospital, Birtinya, Australia; 8 Department of Radiology, Princess Alexandra Hospital, Brisbane, Australia; 9 Department of Renal Medicine, Westmead Hospital, Sydney, Australia; 10 Osteoporosis and Bone Biology Division, Garvan Institute of Medical Research, Sydney, Australia

**Keywords:** Aortic calcification, Arterial stiffness, Chronic kidney disease, Fibroblast growth factor 23, Soluble klotho

## Abstract

**Background:**

Arterial stiffness is an independent predictor of all**-**cause and cardiovascular mortality in patients with chronic kidney disease (CKD). There are limited prospective data however on progression of arterial stiffness in CKD, including evaluating associations with bone mineral markers such as fibroblast growth factor 23 (FGF23) and soluble α-klotho (sKl).

**Methods:**

In this prospective, single-center, observational study, arterial stiffness [measured by pulse wave velocity (PWV)] and hormones influencing mineral homeostasis, including serum FGF23 and sKl, were compared between non-dialysis CKD stages 4/5 and healthy controls at baseline and 12 months (12 m). Abdominal aortic calcification (AAC) was quantitated using lateral lumbar radiography at baseline.

**Results:**

Forty patients with CKD [mean estimated glomerular filtration rate (eGFR) 19.5 ± 6.7 mL/min/1.73m^2^] and 42 controls (mean eGFR 88.6 ± 12.9 mL/min/1.73m^2^) completed follow-up. There were no differences in age, gender and body mass index between groups. A significant increase in FGF23 [240.6 (141.9–1129.8) to 396.8 (160.3–997.7) pg/mL, *p* = 0.001] was observed in the CKD group but serum phosphate, corrected calcium, parathyroid hormone and sKl did not change significantly over 12 m. At baseline, CKD subjects had higher AAC prevalence [83.8% versus (vs.) 43.6%, *p* = 0.002] and higher aortic PWV [9.7(7.6–11.7) vs. 8.1 (7.2–9.7) m/s, *p* = 0.047] compared to controls. At 12 m, aortic PWV increased by 1.3 m/s (95% confidence interval, 0.56 to 2.08, *p* < 0.001) in the CKD cohort, with 30% of subjects showing progression from normal aortic elasticity to stiffening (PWV > 10 m/s). Serum FGF23 was associated with AAC, abnormal PWV and progression of PWV at 12 m.

**Conclusions:**

Arterial stiffness and serum FGF23, both of which are associated with increased cardiovascular risk, increased over one year in individuals with CKD. Additionally, a significant association was found between serum FGF23 and arterial calcification and stiffness. Larger clinical studies and further experimental work are warranted to delineate the temporal relationship as well as the pathological mechanisms linking FGF23 and vascular disease.

**Electronic supplementary material:**

The online version of this article (10.1186/s12882-017-0705-4) contains supplementary material, which is available to authorized users.

## Background

Cardiovascular disease (CVD) is the leading cause of death in patients with chronic kidney disease (CKD) [1]. A major contributor to CVD in this cohort is the elevated burden of both accelerated atherosclerosis and vascular calcification (VC). Approximately 70–80% of patients have evidence of VC at commencement of dialysis [2]. Accelerated atherosclerosis and calcification of both medial and intimal layers of coronary and systemic arteries result in reduced coronary perfusion and increased arterial stiffness, commonly measured using the well-validated methodology of pulse wave velocity (PWV) [3]. Higher PWV in patients with CKD is associated with a substantial increase in all-cause mortality and both fatal and non-fatal cardiovascular events. More importantly, these associations are independent of traditional cardiovascular risk factors [4].

Exploration of novel mechanistic pathways contributing to increased VC and arterial stiffness may provide new therapeutic strategies to modify CVD burden in CKD. Abnormal bone mineral metabolism (BMM) is a key promoter of VC, [5, 6] and this concept is supported by in vitro promotion of calcification and phenotypic changes induced by high phosphate and calcium in vascular smooth muscle cells (VSMC) [6, 7]. More recently, fibroblast growth factor 23 (FGF23) and its co-receptor, klotho, have emerged as key regulators of mineral homeostasis. FGF23, a bone derived hormone, [8] regulates phosphate reabsorption in renal proximal tubules, inhibits production and increases metabolism of 1,25-dihydroxyvitamin D [1,25(OH)_2_D], and is associated with regulation of parathyroid hormone (PTH) by klotho dependent and independent pathways [9–11]. Serum FGF23 levels rise early and exponentially in CKD prior to any detectable increase in serum phosphate and PTH concentrations [12].

Adaptive responses to maintain phosphate homeostasis become ineffective as CKD progresses, and klotho deficiency is likely an early contributor [13, 14]. Klotho influences phosphate flux through the sodium/phosphate co-transporter 2a and 2c, [15] but also exhibits several other protective effects against cell senescence, apoptosis and VC [16]. Up-regulation of FGF23 together with klotho deficiency in animal models has been reported to promote VC and arterial stiffness [17]. Observational human studies have not uniformly demonstrated these associations. Thus, this prospective, observational study aimed to 1) assess changes in FGF23, soluble α-klotho (sKl) and other BMM markers, 2) examine alterations in PWV and 3) assess the clinical correlates of BMM, abdominal aortic calcification (AAC) and change in arterial compliance over 12 months, comparing a cohort of non-dialysis CKD stages 4 and 5 patients to healthy controls.

## Methods

### Study design and setting

A single-center, prospective, longitudinal observational study was conducted. Subjects were recruited from February 2013 to September 2013. All subjects underwent a baseline visit at recruitment and a follow-up visit at 12 months.

### Study population

Subjects aged 18 years or older from the outpatient nephrology clinic at Princess Alexandra Hospital, Brisbane, Australia, were invited to participate in this study if they had stable kidney function for 3 months and an estimated glomerular filtration rate (eGFR) of less than 30 ml/min per 1.73 m^2^. Exclusion criteria included renal replacement therapy, history of solid organ transplantation, treatment with immunosuppression, hormone replacement therapy or chemotherapy in the 12 months prior to enrolment in the study. A healthy control population with normal kidney function and no albuminuria (eGFR >60 ml/min/1.73m^2^, urinary albumin-to-creatinine ratio ≤ 2.5 mg/mmol for males and ≤3.5 mg/mmol for females) was also recruited. A flow chart of study recruitment is shown in Additional file 1: Figure S1. Forty CKD and 42 controls completed follow-up visit at 12 months.

### Clinical assessments

Clinical data, biochemical sampling, physical assessment and vascular imaging were collected and performed on the day of study enrolment (baseline) and at 12-month follow-up visit. Demographic data, including an assessment of risk factor status and current illness, were recorded. All prescribed and non-prescribed medications were documented. Self- reported physical activity was measured using the Active Australia questionnaire [18]. Subjects were classified as physically active if they performed ≥600MET minutes per week [19]. Hypertension and hyperlipidemia were defined by the use of antihypertensive or lipid-lowering therapy, respectively. Diabetes was defined by a history of this diagnosis or use of oral hypoglycemic agents or insulin. Previous CVD was defined as a history of documented myocardial infarction, coronary artery bypass surgery, percutaneous coronary intervention, or hospital admission with acute coronary syndrome (ischemic chest pain and/or electrocardiographic [ECG] changes suggestive of ischemia with no elevation in cardiac enzymes), or peripheral vascular disease including peripheral revascularization procedure or amputation due to ischemia. Subjects had anthropometric assessment, and body mass index (BMI) was calculated as weight divided by the square of the height (kg/m^2^). Blood pressure was the average of three seated measurements taken after a 5-min rest.

### Biochemical assessments

Blood and spot urine samples were obtained in the morning after a minimum 8-h fast. Serum concentrations of creatinine, albumin, urate, calcium (corrected for albumin), phosphate, PTH, 25-hydroxyvitamin D [25(OH) D], 1,25(OH)_2_D, glucose, high sensitivity C-reactive protein (CRP), hemoglobin, and total cholesterol were determined using standard automated laboratory techniques. eGFR was calculated using the Chronic Kidney Disease - Epidemiology Collaboration (CKD-EPI) equation [20]. Random spot urine specimens were collected for assessment of urinary albumin-to-creatinine (uACR) and protein-to-creatinine ratios (uPCR).

Plasma FGF23 levels were measured using the Kainos intact-FGF23 ELISA kit (Kainos Laboratories Inc., Tokyo, Japan) according to the manufacturer’s protocol. Intra-assay and inter-assay analytical coefficients of variation (CV) were 7.3% and 9.9% respectively for samples measured in duplicate. Serum sKl concentrations were measured using the IBL soluble klotho ELISA kit (Immuno-Biological Laboratories Co., Ltd., Gunma, Japan) according to the manufacturer’s protocol with intra-assay and inter-assay analytical CVs 4.1% and 9.3%, respectively, for samples measured in duplicate. All baseline and follow-up samples were measured concurrently.

### Vascular imaging

#### Aortic pulse wave velocity (PWV)

Aortic PWV (m/s) was measured by a single clinician using a non-invasive tonometer (SphygmoCor® 2000; AtCor Medical, Sydney, Australia) placed over the carotid and femoral arteries at rest. Pressure signals were calibrated using brachial blood pressure and measurements were taken of the distance of the carotid and femoral pulses from a fixed point (the suprasternal notch). PWV was calculated using the foot-to-foot method, gated to the cardiac cycle using a 3-lead electrocardiograph [21]. A validated cut-off value of 10 m/s was used to discriminate normal arterial elasticity and arterial stiffness [22].

#### Lateral lumbar radiography

Thirty-six CKD and 36 control subjects agreed to undergo additional evaluation with lateral lumbar radiography. Abdominal aortic calcification (AAC) score was calculated using a validated 24 points system [23] by a radiologist blinded to group and clinical history. Calcified deposits at the anterior and posterior wall in the abdominal aorta were determined at each lumbar vertebra (L1 to L4) and were given a severity score from 0 to 4 (0: no aortic calcified deposits; 1: small calcified deposits and <1/3 of longitudinal aortic wall; 2: moderate calcified deposits ≥1/3 but less than 2/3 of longitudinal aortic wall; 3: severe calcified deposits with ≥2/3 of longitudinal aortic wall). Presence of aortic calcification was defined as total AAC score ≥ 1.

### Statistical analysis

Descriptive statistics were used to represent characteristics between the CKD and control groups at the study entry. Results were expressed as frequencies and percentages for categorical variables, mean ± standard deviation (SD) for normally distributed variables and median (interquartile range) for non-normally distributed variables. Baseline differences between the groups were analyzed by chi-square test for categorical data, unpaired t-test for continuous normally distributed data and Wilcoxon-Mann Whitney test for continuous non-normally distributed data. PWV, FGF23, PTH and sKl were log transformed. Associations between AAC and PWV and predictor variables of interest were assessed using Pearson correlation coefficients for continuous normally distributed variables and Spearman’s correlation for categorical or non–normally distributed data.

Changes in parameters over time were assessed by comparison between baseline and follow-up data using paired t-test and Wilcoxon signed ranks test as appropriate. Multivariate linear regression was used to evaluate the associations between bone parameters and AAC score, PWV at 0 and PWV at 12 months. In addition, logistic regression was used to evaluate the predictors of the following binary measures; AAC and abnormal PWV in the entire study population. Model diagnostics were performed for all linear regression models. Data were analyzed using standard statistical software (Stata 13; http://www.stata.com). *P* values of less than 0.05 were considered statistically significant for all described analyses.

## Results

### Baseline characteristics

Baseline characteristics of the 40 CKD cases and 42 control subjects who completed the study are presented in Table 1. No significant differences were observed between the groups for age, gender, BMI, racial origin, blood pressure, use of cholecalciferol and calcium supplements. Compared with controls, CKD subjects were more likely to receive activated vitamin D, angiotensin converting enzyme inhibitors (ACEi) and to have co-morbidities including diabetes mellitus, hypertension, CVD and hyperlipidemia. CKD patients also had significantly lower levels of serum albumin, 1,25(OH)_2_D, hemoglobin and serum bicarbonate and higher serum levels of urate, calcium, phosphate, PTH and plasma FGF23.

**Table 1:** Baseline characteristics of 40 CKD subjects and 42 controls

Variables	CKD (*n* = 40)	Controls (*n* = 42)
Age (years)	62.9 ± 10.2	59.9 ± 9.8
Male (%)	28 (70.0)	26 (62.0)
BMI (kg/m^2^)	28.5 ± 5.3	27.6 ± 4.8
Race-Caucasian (%)	37 (95)	39 (95)
Diabetes mellitus (%)	17(42.5)^**^	4(9.5)
CVD (%)	12 (30.8)^*^	3 (7.1)
Hypertension (%)	38 (95)^**^	14 (33)
Hyperlipidemia (%)	26 (65)	13 (31)
Physically active (%)	18 (45)^*^	30 (73)
Systolic BP (mmHg)	131 ± 16	133 ± 16
Diastolic BP (mmHg)	75 ± 9	79 ± 10
eGFR (ml/min/1.73m^2^)	19.5 ± 6.7^**^	88.6 ± 12.9
uPCR (mg/mmol)^a^	84 (36–154)^**^	7 (5–9)
uACR (mg/mmol)^a^	43 (8.4–101.5)^**^	0.6 (0.5–0.9)
Serum Albumin (g/L)	38.5 ± 3.6^**^	41.5 ± 2.6
Hemoglobin (g/L)	120.7 ± 16.0^**^	145.0 ± 11.1
Urate (mmol/L)	0.43 ± 0.09^**^	0.33 ± 0.09
Serum bicarbonate (mmol/L)	23.4 ± 2.2^**^	27.1 ± 2.4
CRP (mg/L)^a^	1.0 (1.0–5.6)	1.0 (1.0–3.1)
Serum Corrected Calcium (mmol/L)	2.37 ± 0.14^*^	2.29 ± 0.07
Serum Phosphate (mmol/L)	1.40 ± 0.24^**^	1.13 ± 0.19
PTH (pmol/L)^a^	11.0 (7.2–22.0)^**^	3.5 (2.8–4.3)
25(OH) D (nmol/L)	89.8 ± 29.7^*^	77.6 ± 21.9
1,25(OH)_2_D (nmol/L)	83.9 ± 57.9^**^	145.4 ± 40.5
Soluble α-klotho (pg/mL)^a^	576.7 (473.6–704.3)	613.9 (497.3–933.8)
FGF23 (pg/mL)^a^	240.6 (141.9–1129.8)^**^	46.8 (33.1–56.3)
PWV (m/s)^a^	9.7 (7.6–11.7)^*^	8.1 (7.2–9.7)
AAC (%)	31 (83.8)^*^	17 (48.6)
AAC score	8 (3–14)	1 (0–5)
Medication Use		
Cholecalciferol (%)	8 (20.0)	4 (9.5)
Activated vitamin D (%)	16 (38.1)^**^	0 (0)
Calcium Supplements (%)	7 (17.5)	4 (9.5)
ACEi/ARB (%)	28 (70.0)^**^	11 (26.2)
F/up duration (months)	12.4 ± 0.8	12.3 ± 0.7

Data presented as mean ± standard deviation, numbers (%) or median (interquartile range); ^a^using non-parametric Wilcoxon signed rank sum test

^*^denotes *p* < 0.05

^**^denotes *p* ≤ 0.001 compared to controls

*Abbreviations*: *1,25(OH)*_*2*_ *D* 1,25 dihydroxyvitamin D, *25(OH)D* 25 hydroxyvitamin D, *AAC* abdominal aortic calcification, *ACEi/ARB* angiotensin converting enzyme inhibitor/angiotensin receptor blocker, *BMI* body mass index, *BP* blood pressure, *CI* confidence interval, *CRP* C-reactive protein, *CVD* cardiovascular disease, *eGFR* estimated glomerular filtration rate, *FGF23* fibroblast growth factor 23, *PTH* parathyroid hormone, *PWV* pulse wave velocity, *uACR* urinary albumin-to-creatinine ratio, *uPCR* urinary protein-to-creatinine ratio

**Table 2:** Clinical correlates of a) presence of aortic calcification and b) total abdominal aortic calcification (AAC) score in entire study population

	Unadjusted OR (95% CI)	*p*	Adjusted OR (95% CI)^a^	*p*
a) Presence of aortic calcification (AAC ≥ 1)				
Age (year)	1.23 (1.12–1.36)	<0.001	1.31 (1.12–1.52)	0.001
Log FGF23 (pg/ml)	2.18 (1.28–3.71)	0.004	7.43 (1.07–51.8)	0.04
	Unadjusted Coefficient (95% CI)	*p*	Adjusted Coefficient (95% CI)^a^	*p*
b) AAC score				
Age (year)	0.33 (0.20–0.47)	<0.001	0.21 (0.09–0.34)	0.001
Diabetes mellitus	5.66 (2.62–8.69)	<0.001	4.2 (1.42–6.97)	0.004
Log FGF23 (pg/ml)	2.35 (1.38–3.32)	<0.001	2.61 (1.41–6.98)	<0.001

*Abbreviations*: *AAC* abdominal aortic calcification, *CI* confidence interval, *FGF23* fibroblast growth factor 23, *OR* odds ratio

^a^Adjusted for age, diabetes, hypertension, eGFR, corrected calcium, phosphate, 1,25(OH)_2_D, FGF23 and Klotho

**Table 3:** Clinical correlates of a) baseline abnormal PWV (≥10 m/s); b) baseline PWV and c) 12-month PWV in the entire study population

	Unadjusted OR (95% CI)	*p*	Adjusted OR (95% CI)^a^	*p*
a) Abnormal PWV (≥10 m/s) at baseline				
Age (year)	1.12 (1.05–1.19)	0.001	1.14 (1.04–1.25)	0.004
Diabetes	3.75 (1.33–10.56)	0.01	9.73 (1.37–69.0)	0.02
Log FGF23 (pg/mL)	1.67 (1.14–2.43)	0.007	2.95 (1.04–8.34)	0.04
Log PTH (pg/mL)	1.79 (1.03–3.08)	0.04	3.78 (1.31–10.9)	0.01
eGFR(ml/min/1.73m^2^)	0.99 (0.97–1.00)	0.07	1.08 (1.03–1.15)	0.004
	Unadjusted Coefficient (95% CI)	*p*	Adjusted Coefficient (95% CI)^a^	*p*
b) Baseline PWV				
Age (year)	0.02 (0.01–0.02)	<0.001	0.01 (0.01–0.02)	<0.001
Hypertension	0.25 (0.14–0.37)	<0.001	0.24 (0.16–0.37)	<0.001
eGFR(ml/min/1.73m^2^)	−0.002 (−0.003–0.0004)	0.06	0.006 (0.003–0.008)	<0.001
Log PTH (pg/mL)	0.08 (0.02–0.2)	0.02	0.13 (0.06–0.20)	<0.001
Log FGF23 (pg/mL)	0.06 (0.01–0.10)	0.01	0.05 (−0.003–0.11)	0.06
c) 12-month PWV				
Baseline PWV (m/s)	0.73 (0.56–0.90)	<0.001	0.49 (0.31–0.68)	<0.001
Diabetes	0.18 (0.08–0.29)	0.001	0.16 (0.06–0.26)	0.002
uPCR (mg/mmol)	0.0007 (0.0003–0.001)	0.002	0.0006 (0.0001–0.001)	0.008
Log FGF23 (pg/mL)	0.06 (0.03–0.10)	0.001	0.04 (0.005–0.07)	0.03

^a^Adjusted for age, diabetes, hypertension, eGFR, uPCR, corrected calcium, phosphate, PTH and FGF23

*Abbreviations*: *CI* confidence interval, *eGFR* estimated glomerular filtration rate, *FGF23* fibroblast growth factor 23, *OR* odds ratio, *PTH* parathyroid hormone level, *PWV* pulse wave velocity, *UPCR* urinary protein-to-creatinine ratio

### Comparison of biochemical parameters at baseline and 12-month

At baseline, eGFR strongly correlated with plasma FGF23 (rho = −0.78, *p* < 0.001) but not with sKl (rho = 0.12,*p* = 0.3). Similarly, serum phosphate (rho = 0.56, *p* < 0.001), calcium (rho = 0.34, *p* = 0.002) 25(OH)D (rho = 0.31, *p* = 0.004), 1,25(OH)_2_D (rho = −0.58,*p* < 0.001) and PTH (rho = 0.57,*p* < 0.001) were associated with FGF23 levels but not with sKl.

**Fig. 1:**
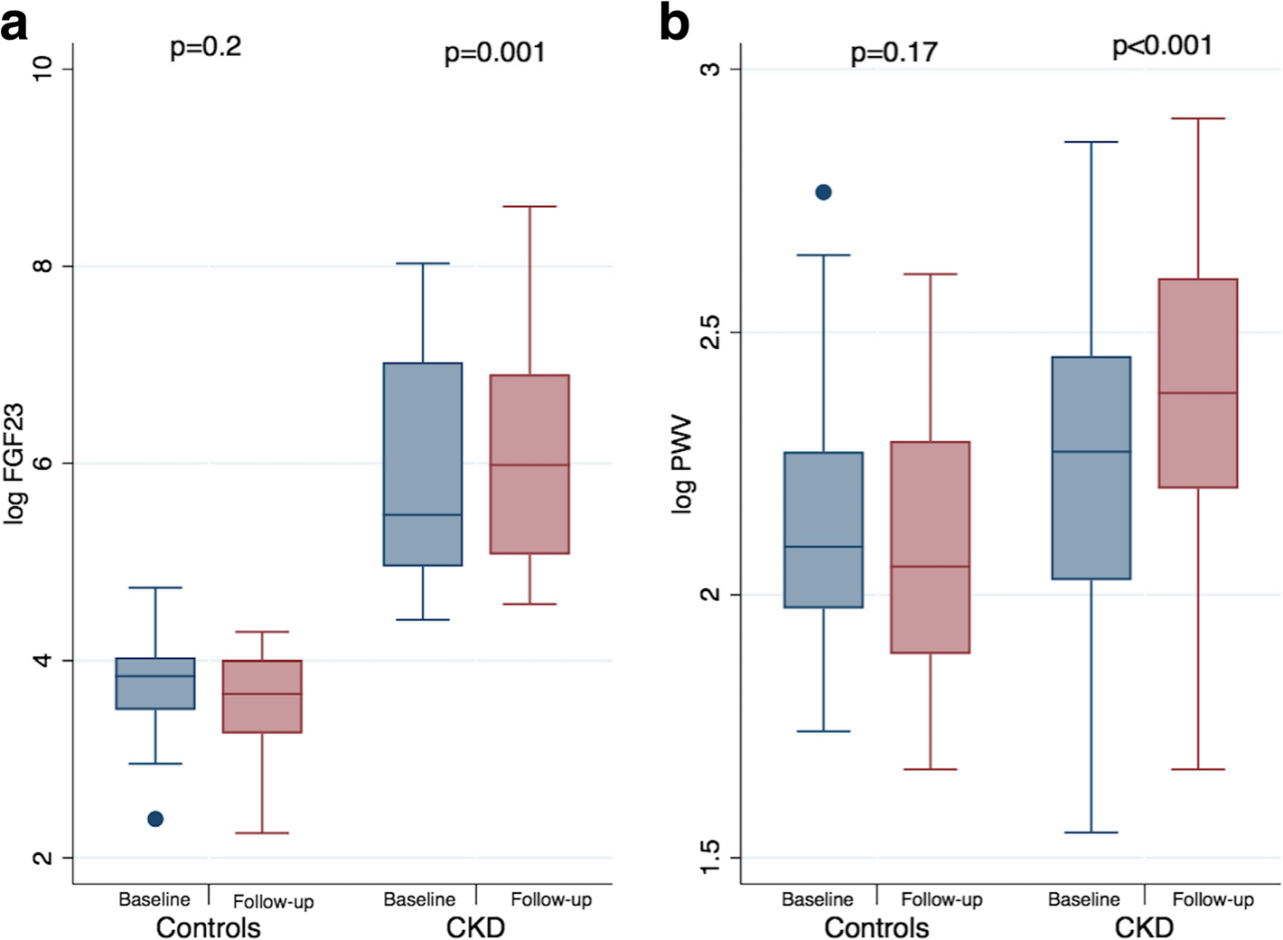
(**a**) Fibroblast growth factor 23 levels and (**b**) pulse wave velocity at baseline and follow-up in CKD and controls. Abbreviations: CKD: chronic kidney disease; FGF23: fibroblast growth factor 23; PWV: pulse wave velocity; p denotes within group comparison at baseline and follow-up

At 12-month follow-up, a significant decline in eGFR [19.5 ± 6.7 to 16.5 ± 6.4 mL/min/1.73m^2^ *p* < 0.001] and a concomitant increase in uPCR [84(36–154) to 130(51–187) mg/mmol *p* = 0.02] were observed only in the CKD group. Three patients commenced dialysis within the one-year study period. A significant increase in FGF23 was seen in the CKD subjects [240.6(141.9–1129.8) to 396.8(160.3–997.7) pg/mL *p* = 0.001] compared to controls (Fig. 1a). The control group had an increase in 1,25(OH)_2_D (145 ± 40 to 175 ± 60 nmol/L, *p* = 0.002). No other significant changes were observed over the 12-month period for other BMM markers and biochemical parameters assessed including sKI and PTH, blood pressure measurements or medication use for both groups (Additional file 2: Table S1).

### Abdominal aortic calcification

**Fig. 2:**
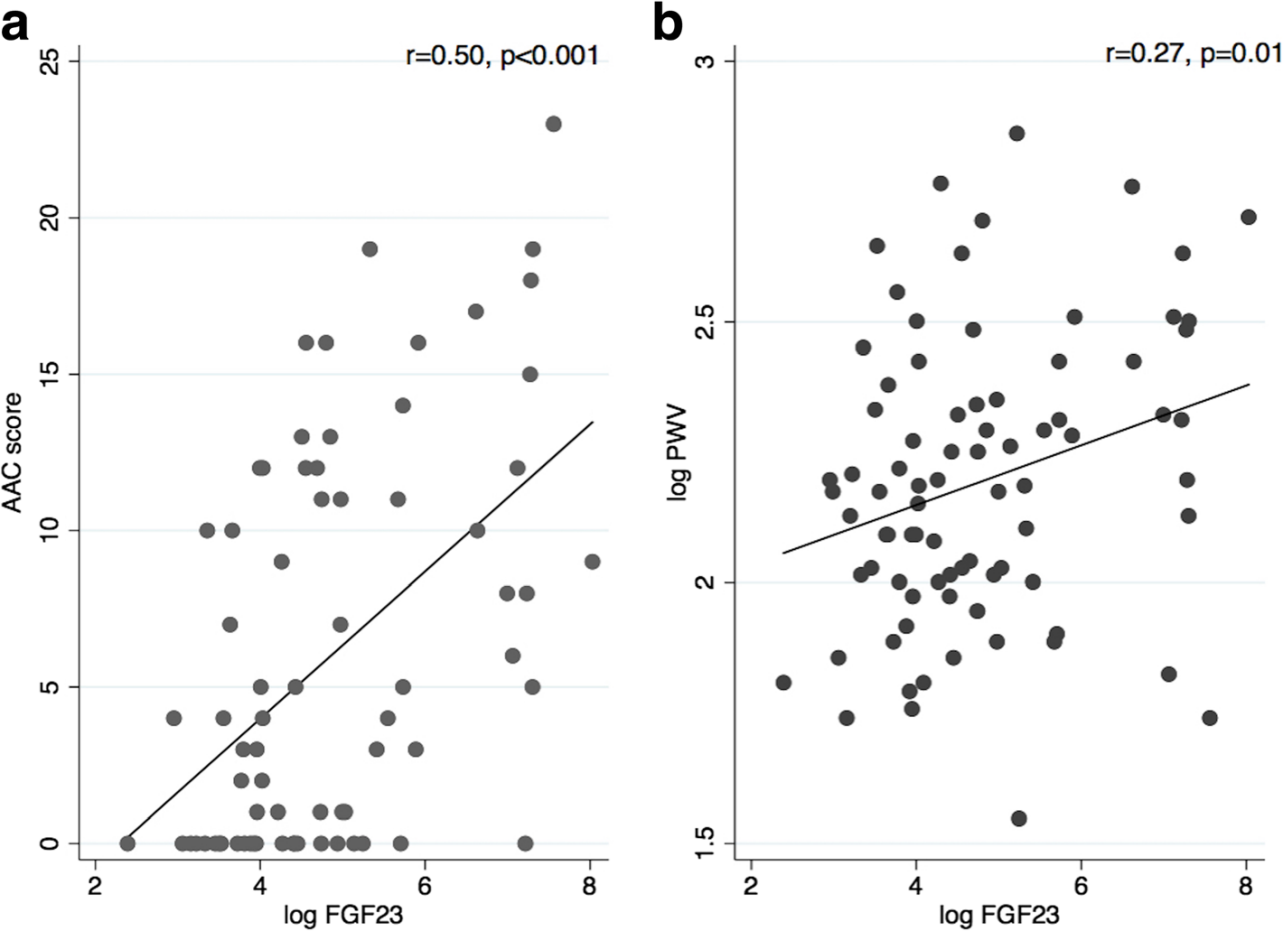
Correlations between fibroblast growth factor 23 and (**a**) abdominal aortic calcification score and (**b**) pulse wave velocity at baseline for the total study population. Abbreviations: AAC: abdominal aortic calcification; FGF23: fibroblast growth factor 23; PWV: pulse wave velocity

The prevalence of AAC was 83.8% among CKD subjects with a significantly higher AAC score compared to controls [median AAC score; CKD 8(3–14) versus (vs.) controls 1(0–5), *p* < 0.001] (Table 1). Bone mineral parameters that correlated with AAC score were calcium (rho = 0.37, *p* = 0.001), phosphate (rho = 0.32, *p* < =0.006), 1,25(OH)_2_D (rho = −0.24,*p* = 0.04), FGF23(rho = 0.50, *p* < 0.001 Fig. 2a) and sKl levels (rho = −0.36, *p* = 0.002). Presence of aortic calcification and higher AAC score were independently associated with higher FGF23 [odds ratio (OR): 7.43 95% confidence interval (CI) 1.07–51.8 for AAC ≥ 1 and adjusted coefficient of 2.61 95%CI 1.21–4.01 for AAC score] in the entire study cohort (Table 2).

### Baseline and follow-up PWV

At baseline, PWV readings were higher in the CKD group compared to controls. [CKD: 9.7(7.6–11.7) versus (vs.) controls: 8.1(7.2–9.7) m/s, *p* = 0.047, Table 1]. Forty three percent of CKD patients compared to 26.2% of controls had arterial stiffness with PWV ≥ 10 m/s (*p* = 0.1). In univariate analysis, FGF23 (Fig. 2b), PTH, phosphate and corrected calcium significantly correlated with baseline PWV. Following adjustment, PTH was independently associated with baseline PWV. Using logistic regression, every 1 pg/mL increase in log transformed FGF23 and PTH were associated with 2.9- and 3.8- fold higher odds of abnormal PWV (≥10 m/s) respectively (Table 3) in all participants.

Aortic PWV significantly increased by 1.3 m/s (95% confidence interval, 0.56 to 2.08, *p* < 0.001) among CKD subjects but did not significantly change in controls after one year (Fig. 1b). This equates to an increase of 16.7% from baseline PWV among CKD subjects. In addition, 30% of CKD subjects progressed from normal arterial elasticity to stiffness compared to only 7% in the controls (*p* = 0.006). Following adjustment for the total study population, only diabetes, higher baseline PWV and higher FGF23 remained independent predictors of PWV at 12 months (Table 3).

## Discussion

This prospective one-year study identified significant increases in PWV and FGF23 levels in CKD stages 4/5 but no significant change was observed in sKl. In addition, the current study demonstrated robust associations between FGF23 and both AAC and progression of arterial stiffness.

PWV is an established predictor of CV and all-cause mortality in both the general and populations [22]. Every 1 m/s increase in PWV is associated with a 15% increase in mortality in the general population [24]. The risk has been reported to double in patients with end-stage kidney disease (ESKD) [25]. A 15% increase in PWV over a 12-month period has previously been described in patients undergoing maintenance peritoneal dialysis [26]. However, the progression of PWV in non-dialysis dependent advanced CKD is not well characterized. One recent study showed an increase in PWV by 1.1 m/s over one-year in 70 patients with mild to moderate CKD [27]. The study presented here not only revealed a similar 1.3 m/s (16.7%) increase in PWV in advanced CKD (stages 4/5) but also demonstrated that such progression in PWV measurements was limited to CKD subjects and not seen in healthy controls.

Prior studies have evaluated the associations between FGF23 and arterial stiffness with inconsistent outcomes. These studies have been cross-sectional in design and lacked homogeneity in assessment of arterial stiffness. One study demonstrated an independent association between intact-FGF23 and pulse wave analysis among 967 elderly patients, that was more pronounced among patients with renal impairment [28]. In contrast, using ankle brachial index in 5977 participants with a mean eGFR of 84 ± 17 mL/min/1.73m^2^, Hsu et al. did not find an independent association between intact-FGF23 and small/large arterial elasticity. Nonetheless, these investigators established that the highest decile of FGF23 correlated with larger pulse pressure suggesting an association with poorer vascular compliance [29]. In the present longitudinal study, the positive association between FGF23 and abnormal PWV at baseline and progression of PWV at 12-month were found in all participants independent of underlying renal function suggesting that FGF23 may have a substantial role in mediating arterial stiffness.

The association between FGF23 and VC also lacks consistency in the current literature. The Chronic Renal Insufficiency Cohort (CRIC) investigators reported no association between intact-FGF23 levels and coronary/thoracic artery calcium in 1501 patients with earlier stages of CKD (mean eGFR of 47 ± 17 mL/min/1.73 m^2^), although this study was largely limited by a lag time of up to two years between the measurement of FGF23 and vascular imaging [29]. This differs from the findings by Desjardins et al. who reported that plasma intact-FGF23 levels were independently correlated with aortic calcification scores in 142 patients with a wider spectrum of renal dysfunction (CKD 2–5) inclusive of patients on maintenance dialysis [30]. Intact- FGF23 was also found to correlate with progression of coronary artery calcification over the course of a year among 74 hemodialysis patients [31]. The study presented here strengthens the previously reported association between FGF23 concentration and aortic calcification. A novel aspect of this study was the comprehensive assessment of the association of arterial calcification with bone mineral markers including FGF23, sKl, phosphate and PTH.

Numerous mechanisms including direct or indirect pathways have been proposed to explain the link between FGF23 and vascular disease. Firstly, severe widespread calcification is well-characterized in klotho-knockout mice models that also develop markedly elevated FGF23 levels [32, 33]. FGF23 has been shown to not only directly induce endothelial injury by reducing nitric oxide (NO) metabolites and inhibiting endothelium-dependent aortic vasodilatation, [34] but also increased intracellular calcium and altered myocardial contractility [35]. Furthermore, intramyocardial administration of FGF23 resulted in left ventricular hypertrophy in the absence of klotho [36]. In contrast, FGF23-driven osteoblastic differentiation of VSMC was only reported in the presence of klotho suggesting the FGF23 effect on the vasculature is klotho-dependent [17]. Meanwhile, Shalhoub et al. demonstrated that neutralization of FGF23 with a targeted monoclonal antibody induced hyperphosphatemia and this was detrimental to the rat model resulting in greater aortic calcification [37]. It is also possible that high levels of FGF23 may have actions independent to other bone mineral markers including regulation of renin angiotensin system and chronic inflammation [38]. In CKD mouse models, FGF23 was found to suppress angiotensin-converting enzyme 2 and activates lipocalin-2, transforming growth factor-beta and tumor necrosis factor-alpha [39]. Taken together, the link between FGF23 and vascular disease seems clear though whether FGF23 is a direct vascular toxin or promotes vascular injury through regulation of phosphate in a klotho-dependent manner exclusively is yet to be confirmed. In addition, the inconsistent reporting of the association between VC and FGF23 may indicate variability in the commercially available assays and more work is required in this area [40].

Animal models and in vitro studies have found klotho to have a direct vasculo-protective effect [41–43]. However, there are limited human studies to support this plausible relationship. sKl was reported to be significantly lower in patients who had significant coronary artery disease (CAD) diagnosed on coronary angiography compared to individuals without significant disease (total cohort of 371 patients) [44]. Notably, reduced vascular expression of klotho mRNA was found to be associated with severity of CAD in patients without renal impairment [44]. Additionally, decreased sKl concentration was associated with arterial stiffness but not with endothelial dysfunction or VC in 114 patients with moderate CKD [45]. However, the relationship between sKl and aortic calcification scores seen in this study was lost, in particular, following adjustment for FGF23. Findings in our study suggest FGF23 may have a greater association with vascular burden compared to sKl. Further studies, both clinical and experimental, examining FGF23 and sKl concurrently are required for thorough understanding of their concomitant roles in accelerated vascular disease.

The findings from this study warrant careful interpretation due to some limitations; 1) the relatively small sample size in both groups and the single-center design potentially limited the generalizability of the study’s findings; 2) progression of AAC was not assessed as follow-up lumbar X-ray was not obtained; 3) a cause-effect relationship could not be identified due to the observational nature of the study; 4) several co-existing conditions, such as underlying diabetes, CVD and physical inactivity, that were prevalent in the CKD group may have confounded the observed associations in this study; 5) even though a large number of patient characteristics (including diabetic status) were adjusted for, the possibility of residual confounding cannot be excluded; 6) information on dietary phosphate intake and urinary phosphate excretion, which may directly influence change in FGF23 and sKl, was not collected in this study and 7) evaluation of cardiac function and structure was also not collected in this study and may have provided valuable information on the link between FGF23 and cardiovascular disease.

## Conclusions

FGF23 and PWV were observed to substantially increase in CKD subjects compared to controls over a year. Elevated circulating FGF23 levels were associated with aortic calcification and progression of arterial stiffness independent of other BMM markers evaluated in this study in both CKD and controls. These findings should be confirmed in large, prospective studies and in those with earlier stages of CKD. Causal relationships should be explored further in vitro with a view to interrogating mechanistic pathways and seeking potential therapies that may be useful in reducing the vascular burden in CKD.

## Additional files

Additional file 1: Figure S1. Flow diagram of recruitment. (DOCX 35 kb)

Additional file 2: Table S1. Changes in bone mineral markers, biochemical variables, blood pressure and medications over 1 year in CKD and controls. (DOCX 15 kb)

## Abbreviations

1,25(OH)_2_D: 1,25 dihydroxyvitamin D
25(OH)D: 25 hydroxyvitamin D
AAC: Abdominal aortic calcification
ACEi: Angiotensin converting enzyme inhibitor
ARB: Angiotensin receptor blocker
BMI: Body mass index
BP: Blood pressure
CAD: Coronary artery disease
CI: Confidence interval
CKD: Chronic kidney disease
CKD-EPI: Chronic Kidney Disease- Epidemiology Collaboration
CRP: C-reactive protein
CV: Coefficients of variation
CVD: Cardiovascular disease
ECG: Electrocardiographic
eGFR: Estimated glomerular filtration rate
ESKD: End-stage kidney disease
FGF23: Fibroblast growth factor 23
OR: Odds ratio
PTH: Parathyroid hormone
PWV: Pulse wave velocity
sKI: soluble α-klotho
uACR: urinary albumin-to-creatinine ratio
uPCR: urinary protein-to-creatinine ratio
VC: Vascular calcification
VSMC: Vascular smooth muscle cells
