# Supplementary material for: Progression of arterial stiffness is associated with changes in bone mineral markers in advanced CKD
Source: BMC Nephrol. 2017 Sep 4;18:281. doi: 10.1186/s12882-017-0705-4 (PMC5584006; doi:10.1186/s12882-017-0705-4)
Supplement: Supplementary file 1 — Flow diagram of recruitment. (DOCX 35 kb) [file 12882_2017_705_MOESM1_ESM.docx]

Figure S1: Flow diagram of recruitment

**Assessed for eligibility n= 159 CKD patients,**

**n=50 healthy controls**

**Inclusion Criteria :**

- eGFR≤ 30 mL/min/1.73m^2^,age ≥18, not on dialysis (for CKD)

**Exclusion Criteria**

- eGFR>30 mL/min/1.73m^2^ (for CKD)
- treatment with bisphosphonate, cyclophosphamide, cyclosporine, androgen, oestrogen replacement, anti-epileptic, chemotherapy, glucocorticoid
- known osteoporosis
- Organ transplant recipient

Total recruitment: CKD (n=50) and controls (n=47)

1. *CKD excluded due to out of range eGFR*
2. *Controls excluded due to albuminuria*

CKD 47: CONTROLS 45 at baseline

***7 CKD drop-outs:***

*-1 died*

*- 1 commenced chemotherapy*

*- 1 moved interstate*

*- 1 commenced prednisolone*

*- 1 lost to follow up*

*- 2 prolonged hospitalization*

***3 Control drop-outs:***

*- 2 moved interstate*

*- 1 lost to follow up*

CKD 40: CONTROLS 42 at 12 months follow up visit
