# Supplementary material for: Progression of arterial stiffness is associated with changes in bone mineral markers in advanced CKD
Source: BMC Nephrol. 2017 Sep 4;18:281. doi: 10.1186/s12882-017-0705-4 (PMC5584006; doi:10.1186/s12882-017-0705-4)
Supplement: Supplementary file 2 — Changes in bone mineral markers, biochemical variables, blood pressure and medications over 1 year in CKD and controls. (DOCX 15 kb) [file 12882_2017_705_MOESM2_ESM.docx]

**Table S1:** Changes in bone mineral markers, biochemical variables, blood pressure and medications over 1 year in CKD and controls

| **Parameter** | **CKD** | | | **Control** | | |
| --- | --- | --- | --- | --- | --- | --- |
|  | **Baseline**  **(n=40)** | **Follow-up**  **(n=40)** | **p** | **Baseline**  **(n=42)** | **Follow-up**  **(n=42)** | **p** |
| Klotho (pg/mL) | 576.7 (473.6-704.3) | 555.0 (440.0- 724.2) | 0.2 | 613.9 (497.3-933.8) | 618.8 (411.1-914.3) | 0.2 |
| Phosphate (mmol/L) | 1.40±0.24 | 1.42±0.22 | 0.4 | 1.13±0.19 | 1.14±0.15 | 0.8 |
| Corrected calcium (mmol/L) | 2.37±0.14 | 2.31±0.2 | 0.08 | 2.29±0.07 | 2.28±0.08 | 0.3 |
| PTH (pmol/L) | 11.0 (7.2-22.0) | 15 (7.8-25.0) | 0.4 | 3.5 (2.8-4.3) | 4.2 (3.0-5.0) | 0.2 |
| 25(OH)D  (nmol/L) | 89.8±29.8 | 82.8±38.5 | 0.09 | 77.6±21.9 | 74.9±19.4 | 0.4 |
| 1,25(OH)_2_D | 83.9±58.7 | 74.7±31.0 | 0.3 | 145.3±40.4 | 175.1±60.5 | 0.002 |
| Urate (mmol/L) | 0.43±0.09 | 0.44±0.12 | 0.6 | 0.33±0.09 | 0.32±0.07 | 0.3 |
| Albumin (g/L) | 38.5±3.6 | 38.7±4.0 | 0.4 | 41.5 ± 2.6 | 41.9±2.7 | 0.4 |
| Serum bicarbonate (mmol/L) | 23.4±2.2 | 23.2±3.2 | 0.6 | 27.1±2.4 | 27.2±0.3 | 0.7 |
| Systolic BP (mmHg) | 131±16 | 129±16 | 0.4 | 133±16 | 134±14 | 0.9 |
| Diastolic BP (mmHg) | 75±9 | 75±12 | 0.9 | 79±10 | 79±10 | 0.9 |
| ACEi/ARB (%) | 28 (70) | 25 (63) | 0.2 | 11 (26) | 13 (31) | 0.2 |
| β-Blocker (%) | 19 (48) | 19 (48) | 1.0 | 4 (10) | 4 (10) | 1.0 |
| Calcium supplements (%) | 7 (18) | 6 (15) | 0.3 | 4 (10) | 4 (10) | 1.0 |
| Activated vitamin D (%) | 16 (38) | 15 (38) | 0.3 | 0 (0) | 0 (0) | 1.0 |
| Cholecalciferol (%) | 8 (20) | 8 (20) | 1.0 | 4 (10) | 6 (14) | 0.2 |

Data presented as mean± standard deviation, number (%) or median (interquartile range)

Abbreviations: 1,25(OH)_2_D: 1,25 dihydroxy vitamin D; 25(OH) D: 25 hydroxy vitamin D; ACEi: angiotensin converting enzyme inhibitor;

ARB: angiotensin receptor blocker; BP: blood pressure
